# *Escherichia coli* Cells Exposed to Lethal Doses of Electron Beam Irradiation Retain Their Ability to Propagate Bacteriophages and Are Metabolically Active

**DOI:** 10.3389/fmicb.2018.02138

**Published:** 2018-09-10

**Authors:** Anne-Sophie Charlotte Hieke, Suresh D. Pillai

**Affiliations:** ^1^National Center for Electron Beam Research (an IAEA Collaborating Centre for Electron Beam Technology), Texas A&M University, College Station, TX, United States; ^2^Interdisciplinary Faculty of Toxicology, Texas A&M University, College Station, TX, United States

**Keywords:** electron beam, ionizing radiation, bacteria, DNA damage, bacteriophages

## Abstract

Reports in the literature suggest that bacteria exposed to lethal doses of ionizing radiation, i.e., electron beams, are unable to replicate yet they remain metabolically active. To investigate this phenomenon further, we electron beam irradiated *Escherichia coli* cells to a lethal dose and measured their membrane integrity, metabolic activity, ATP levels and overall cellular functionality via bacteriophage infection. We also visualized the DNA double-strand breaks in the cells. We used non-irradiated (live) and heat-killed cells as positive and negative controls, respectively. Our results show that the membrane integrity of *E. coli* cells is maintained and that the cells remain metabolically active up to 9 days post-irradiation when stored at 4°C. The ATP levels in lethally irradiated cells are similar to non-irradiated control cells. We also visualized extensive DNA damage within the cells and confirmed their cellular functionality based on their ability to propagate bacteriophages for up to 9 days post-irradiation. Overall, our findings indicate that lethally irradiated *E. coli* cells resemble live non-irradiated cells more closely than heat-killed (dead) cells.

## Introduction

Ionizing radiation and its three main sources, electron beam (eBeam), X-rays, and γ (gamma) rays, are cornerstone technologies of the medical device sterilization and food pasteurization industries (Dorpema, 1990; Follett, 2002; Farkas and Mohacsi-Farkas, 2011; Pillai and McElhany, 2011; Pillai and Shayanfar, 2015). The underlying premise is that at appropriate lethal doses of ionizing radiation, the microbial cells are inactivated, in other words, they are unable to multiply. There are a number of reports detailing the response of viruses, bacteria, and protozoa to ionizing radiation (Purdie et al., 1974; Weiss et al., 1974; Daly and Minton, 1995b; Miyahara and Miyahara, 2002; Kimura et al., 2006; Praveen et al., 2013). There are also a number of studies detailing the possible resistance mechanisms of bacterial cells to ionizing radiation (Daly and Minton, 1995a,b, 1996, 1997; Daly et al., 2007; Holloman et al., 2007; Makarova et al., 2007). Studies have also suggested that ionizing radiation causes structural damage to the DNA in the cells (Krasin and Hutchinson, 1977; Hutchinson, 1985; Daly and Minton, 1995a). However, reports in the literature as well as previous studies in our laboratory suggest that irradiated bacterial cells retain residual metabolic and transcriptional activity. For example, Magnani et al. (2009) demonstrated that lethally gamma irradiated *Brucella melitensis* cells had lost their ability to replicate but still possessed metabolic and transcriptional activity. The cells also persisted in macrophages, generated antigen-specific cytotoxic T cells, and protected mice against virulent bacterial challenge (Magnani et al., 2009). Secanella-Fandos et al. (2014) observed that lethally gamma irradiated *Mycobacterium bovis* cells were metabolically active and exhibited similar tumor growth inhibition and induction of cytokines compared to live cells. In our laboratory, we also observed that when *Salmonella* spp. cells were exposed to lethal doses of eBeam irradiation, the cells were no longer able to multiply. However, the cells had intact membranes and retained their surface antigens (unpublished data). The findings that lethally irradiated cells have DNA double strand breaks, yet are metabolically active and have intact membranes, but are unable to multiply present a scientific conundrum.

The overall objective of this study was to characterize the response of *Escherichia coli* cells (K-12 wild-type strain MG 1655) to a lethal dose of eBeam radiation. Specifically, we investigated the structural damage to the cells’ DNA, their membrane integrity, their metabolic activity (electron transport activity and ATP levels) and whether irradiated cells could serve as hosts for bacteriophages λ, T4, and T7. These bacteriophages require the host cell’s machinery to varying degrees to produce progeny phage particles. Phage λ relies completely on the host cell to reproduce, T4 requires specific cellular components of the host cell, and T7 requires the host’s machinery only at the very beginning of infection (Hendrix and Casjens, 2006; Little, 2006; Molineux, 2006; Mosig and Eiserling, 2006). Non-irradiated, live cells, and heat-killed cells were used as positive and negative controls, respectively. The underlying hypothesis was that eBeam irradiated *E. coli* cells retain enough of their cellular structure and function to serve as host cells for bacteriophage propagation, thereby confirming the metabolic activity and viability of lethally eBeam irradiated bacterial cells.

## Materials and Methods

### Preparation and eBeam Irradiation of Bacterial Cultures

Overnight cultures of the *E. coli* K-12 wild-type strain MG 1655 were grown in Luria-Bertani (LB) broth at 35°C in a shaking water bath. The day of the irradiation, log-phase cultures of *E. coli* were prepared by seeding LB broth with the fresh overnight culture at a ratio of 1:100. The culture was allowed to grow at 35°C to an OD_600_ of ca. 0.5 resulting in approximately 1 × 10^8^ colony forming units (CFU)/ml. The log-phase culture was subsequently chilled on ice for 10 min to arrest cell growth. Aliquots of the log-phase culture in LB broth were packaged for eBeam irradiation. In order to comply with the biosafety regulations of Texas A&M University, aliquots of the cell suspensions were placed in heat-sealed double-bagged Whirl Pak bags (Nasco, New York, NY, United States). These heat-sealed bags were then placed inside 95 kPa specimen transport bags (Therapak, Buford, GA, United States).

Previous studies in our laboratory have shown that irradiating cell suspensions in flat plastic bags produced a dose uniformity ratio (DUR) close to 1.0. A DUR of 1.0 indicates complete dose uniformity throughout the sample. Samples were held at 4°C for less than 2 h prior to irradiation and transported on ice in a Saf-T-Pak transport box (Saf-T-Pak, Hanover, MD, United States). Non-irradiated aliquots of the log-phase culture in LB broth were used as a positive control. The positive control samples were packaged the same way as the experimental samples and were transported to the irradiation facility to eliminate possible differences in survival due to transport and handling. Heat-killed cells (70°C for 60 min) were used as a negative control. The eBeam irradiations were carried out at the National Center for Electron Beam Research (NCEBR) at Texas A&M University in College Station, TX, United States using a 10 MeV, 15 kW eBeam linear accelerator. All eBeam irradiations were carried out at ambient temperature (ca. 25°C). Based on a prior dose-response experiment, it was determined that a dose of 7.0 kilo Gray (kGy) was needed to render 1 × 10^8^/ml *E. coli* cells in LB broth replication incompetent (*data not shown*). Thus, samples were irradiated to a lethal target dose of 7.0 kGy by conveying the samples across the incident eBeam. To confirm that the cells had lost their replication capabilities, cells were plated on LB plates and incubated at 37°C for 4 days.

### Membrane Integrity of eBeam Irradiated *E. coli* Cells

We used the LIVE/DEAD^®^ *Bac*Light^TM^ Bacterial Viability Kit (Molecular Probes^®^, Grand Island, NY, United States), a two-color fluorescent dye system, to characterize the membrane integrity of eBeam irradiated cells. The SYTO^®^ 9 green-fluorescent nucleic acid stain can penetrate cells with either intact or damaged membranes. On the other hand, the red-fluorescent nucleic acid stain, propidium iodide, penetrates only cells with damaged membranes. When used in combination, this dye system stains cells with intact membranes green and cells with damaged membranes red.

Following eBeam irradiation and heat treatment, the *E. coli* samples were stored at 4°C in the LB broth they had been treated in and the membrane integrity was examined at the following time points: 0, 4, 24, and 216 h (9 days). The LIVE/DEAD^®^ *Bac*Light^TM^ Bacterial Viability Kit was used according to the manufacturer’s instructions with minor modifications. Briefly, 0.5 ml of the sample were centrifuged for 1 min at RT at maximum speed in a microcentrifuge. The cell pellet was resuspended in 0.5 ml 0.85% sodium chloride (NaCl) solution. 1.5 μl of the dye mixture (equal volume SYTO^®^ 9 and propidium iodide) were added protected from light. The sample was vortexed and incubated for 15 min at RT in the dark. Slides with 10 μl of sample were prepared for fluorescent microscopy. Images were taken immediately with an Olympus BX50 fluorescent microscope with a FITC/Texas Red filter and a 200× magnification.

### Visualization of DNA Double-Strand Breaks in eBeam Irradiated *E. coli* Cells

We used the neutral comet assay, adapted for bacteria, to visualize DSBs under a fluorescent microscope. This assay, also known as single-cell gel electrophoresis, offers direct visualization of DSBs through the appearance of DNA tails or comets. Cells of interest are immobilized in low melting agarose, lysed, and electrophoresed. This allows the DNA to migrate out of the cell in a pattern determined by the extent of DNA damage (Ostling and Johanson, 1984; Lemay and Wood, 1999).

Following eBeam irradiation, the *E. coli* samples were transported to the laboratory on ice and stored at 4°C for 1–2 h until the comet assay could be performed. The neutral comet assay was performed using the Trevigen CometAssay^®^ protocol (Reagent Kit for CometAssay^®^, Catalog # 4250-050-K) with modifications. Briefly, a 50 μl aliquot (1 × 10^7^ cells/ml) of the appropriate bacterial cell suspension (eBeam irradiated, non-irradiated positive control, and heat-killed negative control) was mixed with lysozyme (final conc. 0.5 mg/ml) and RNase A (final conc. 5 μg.ml) prior to adding 500 μl of molten Comet LMAgarose (0.5% low-melting agarose) (Trevigen Inc., Gaithersburg, MD, United States) kept at 37°C. After mixing the sample, a 50 μl aliquot was pipetted onto the CometSlide (Catalog # 4250-050-03, Trevigen Inc., Gaithersburg, MD, United States), resulting in approximately 50,000 cells per sample area. The slides were incubated at 4°C for 10 min in the dark. Following gelling of the agarose disk, the slides were placed in plastic Coplin Jars containing lysis solution [2.5 M NaCl, 100 mM EDTA, 10 mM Tris pH 10, 1% sodium lauroyl sarcosinate, 1% Triton X-100 (added fresh)] and incubated for 1 h at RT. Following cell lysis, slides were placed in an enzyme digestion buffer [2.5 M NaCl, 10 mM EDTA, 10 mM Tris pH 7.4, 1 mg/ml Proteinase K] for 2 h at 37°C. After draining the excess buffer, slides were immersed in pre-chilled 1× electrophoresis buffer [100 mM Tris pH = 9, 300 mM sodium acetate] and incubated for at least 30 min at 4°C; slides may also be stored overnight at this point. Slides were placed in a horizontal electrophoresis unit (Owl; Model B-2) containing fresh 1× electrophoresis buffer and electrophoresed at 1 V/cm for 1 h at RT. The slides were then placed in 1 M ammonium acetate in ethanol for 30 min at RT. DNA precipitation was followed by ethanol dehydration of the agarose. Slides were immersed in absolute ethanol for 1 h at RT and air-dried, followed by 70% ethanol for 15 min at RT and then air-dried. Slides were then stained with 50 μl of freshly prepared SYTO 9 solution (1.25 μM in 0.04% DMSO) for 15 min in the dark. The excess SYTO 9 stain was removed by gently tapping the slide on a KimWipe. Slides were then air-dried for 30 min in the dark, followed by 5 min at 40°C in the dark. Observations were made using an Olympus BX50 fluorescent microscope with a FITC filter and a 1000× magnification. CFU counts were obtained by plating the *E. coli* samples on LB agar and incubating them at 37°C for 4 days.

### Metabolic Activity in eBeam Inactivated *E. coli* Cells

To investigate the metabolic activity in eBeam inactivated cells over time, we chose an assay that uses cellular reducing conditions to monitor metabolic activity/cell health. Resazurin, the active ingredient, is a non-fluorescent compound. Upon entering the cell, it is converted to resorufin, a highly fluorescent compound, via the cell’s reducing environment. Alive and healthy cells have more reducing power than injured/dead cells and will produce a higher fluorescent signal (Squatrito et al., 1995; Nakayama et al., 1997; Rampersad, 2012).

Following eBeam irradiation and heat treatment, the *E. coli* samples were stored at 4°C in the LB broth they had been treated in and the metabolic activity was examined at the following time points: 0, 4, 24, and 216 h (9 days). Metabolic activity was measured with the redox indicator alamarBlue^®^ (Invitrogen, Grand Island, NY, United States) according to the manufacturer’s instructions. Briefly, 10 μl of the alamarBlue^®^ reagent were added to 100 μl of cells (in a black 96-well plate), mixed, and incubated in the dark at 37°C for 1 h. Following the 1-h incubation, the fluorescence was measured with a Perkin Elmer Wallac 1420 VICTOR2^TM^ microplate reader. Two independent experiments were performed.

### ATP Levels in eBeam Inactivated *E. coli* Cells

Since ATP, an indicator of metabolically active cells, can be detected via a bioluminescence assay (Squatrito et al., 1995), we determined the cellular ATP levels with the BacTiter-Glo^TM^ Microbial Cell Viability Assay (Promega, Madison, WI, United States) according to the manufacturer’s instructions with minor modifications. Following eBeam and heat treatment, the *E. coli* samples were stored at 4°C in the LB broth they had been treated in and the ATP levels were examined at the following time points: 0, 4, 24, and 216 h (9 days) by adding 10 μl of the BacTiter-Glo reagent to 10 μl of cells (in a white 384-well plate). Samples were mixed and incubated for 5 min at RT. Following the incubation, the luminescence was measured with a Perkin Elmer Wallac 1420 VICTOR2^TM^ microplate reader. The cellular ATP concentrations were interpolated from a standard curve.

### Bacteriophage Multiplication in eBeam Inactivated *E. coli* Cells

To study the overall cellular functionality of eBeam inactivated cells we tested their ability to propagate bacteriophages. Following eBeam and heat treatment, the *E. coli* samples were kept at 4°C in the LB broth they had been treated in and the overall cellular functionality was determined at the following time points: 0, 4, 24, and 216 h (9 days). One milliliter of each sample was centrifuged for 1 min at RT in a microcentrifuge at maximum speed. The cell pellet was resuspended in 50 μl amended LB broth (5 mM CaCl_2_ and 5 mM MgSO_4_) and 50 μl of the bacteriophage (lambda vir 101, T4D or T7), also in amended LB broth, were added at a multiplicity of infection (MOI) of 0.01 (10^8^ CFU/ml to 10^6^ PFU/ml). The mixture was vortexed and incubated in a 37°C shaking water bath for 24 h. Following the incubation, samples were placed on ice, diluted in amended LB broth and spot plated on LB agar using the top agar overlay method (Adams, 1959). Ten microliters from each dilution (-0 to -8) were spotted to determine the dilutions that would yield countable numbers. LB plates were incubated at 37°C for 16–18 h. Following spot plating, the samples were stored at 4°C overnight and full plate titrations, also using the top agar overlay method, of the appropriate dilutions were performed the next day. LB plates were incubated at 37°C for 16–18 h and then counted for plaque forming units (PFUs). The ability of the *E. coli* cells to replicate (or not) was confirmed by plating survivors on LB agar plates and incubating them at 37°C for 4 days. Two independent experiments were performed.

### Statistical Analysis

Statistical significance (*P*-value <0.05) was determined through pairwise Student’s *t*-tests using the JMP statistical software (version 11).

## Results

### Membrane Integrity of eBeam Inactivated *E. coli* Cells

**FIGURE 1:**
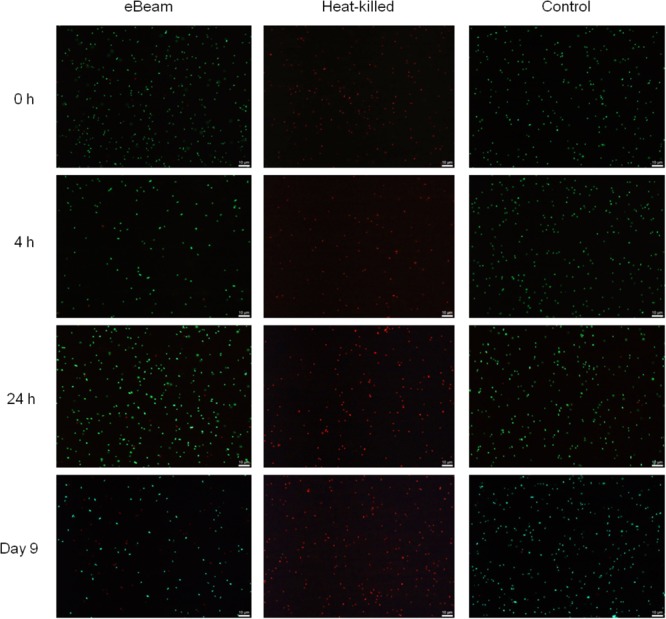
Representative images depicting membrane integrity in eBeam inactivated, heat-killed, and live *Escherichia coli* cells. Cultures were incubated at 4°C in LB broth post-treatment and images were taken at 0, 4, 24 h, and 9 days.

The results indicated that, as expected, the live (non-irradiated) *E. coli* cells had intact membranes at all the time points (**Figure 1**). At both the 24 h and day 9 time points, the live control showed a few cells with damaged membranes. In contrast, the heat-killed cells had only damaged membranes for all the time points (**Figure 1**). Overall, the eBeam inactivated cells had intact membranes similar to the live cells (**Figure 1**). At 0 and 4 h post-irradiation, the eBeam inactivated cultures showed a few cells with damaged membranes. As the incubation continued, the number of cells with damaged membranes increased. At day 9 of incubation in LB broth at 4°C, approximately half of the eBeam inactivated cells showed signs of membrane damage (based on qualitative analysis) (**Figure 1**). These microscopic images are presented without enlargement to highlight the finding that the majority of cells in the field of view are viable (green) and as the incubation proceeds to day 9, the number of cells with compromised membranes (red) increase. Though we unfortunately did not perform quantitative image analysis to quantify the % red and green cells, these microscopic images highlight the intactness of the cellular membrane after eBeam irradiation as compared to the membrane damage that occurs during heating.

### Visualization of DNA Double-Strand Breaks in eBeam Inactivated *E. coli* Cells

**FIGURE 2:**
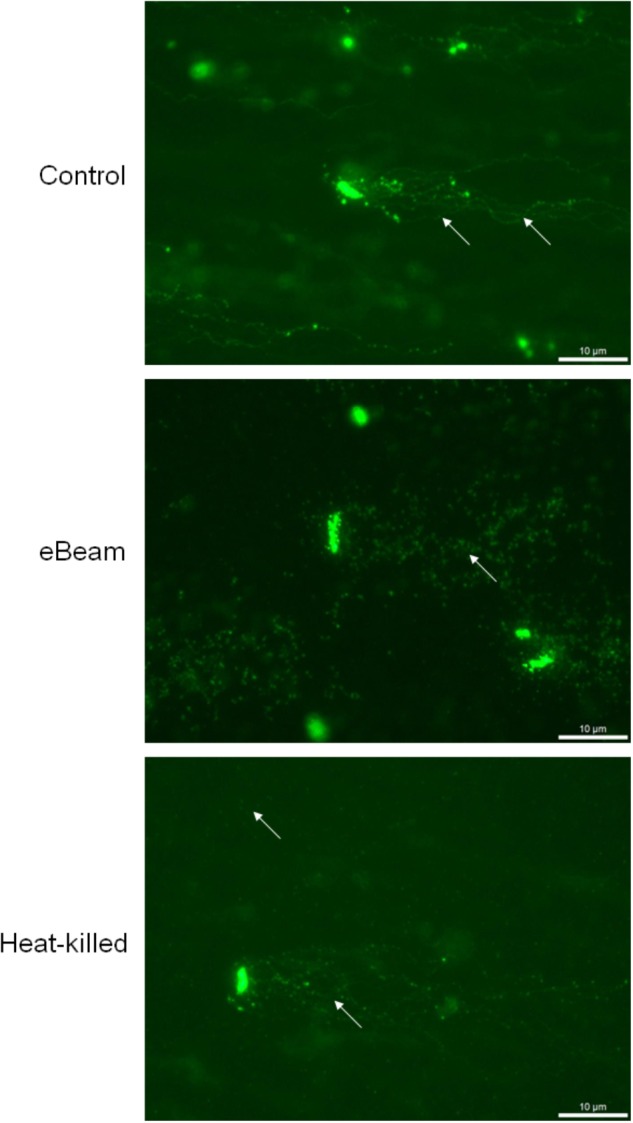
Representative images showing the detection of DNA double-strand breaks in *E. coli* cells using the neutral comet assay. Cells were exposed to either a lethal eBeam irradiation dose (absorbed dose: 7.04 kGy), a lethal heat treatment (70°C for 60 min) or no treatment. Arrows indicate DNA tails (control), putative DNA fragments (eBeam) or both (heat-killed).

The neutral comet assay was performed to visualize the DNA double-strand breaks (DSBs) in *E. coli* cells exposed to a lethal eBeam irradiation dose (7 kGy), a lethal heat treatment (70°C for 60 min) or no treatment (live control). The measured eBeam dose for *E. coli* cells irradiated in LB was 7.04 kGy. The live cells showed only a few DSBs as seen by a few long DNA tails whereas eBeam inactivated cells showed extensive DSBs as seen by no distinct DNA tails. The extent of DNA damage in heat-killed cells was not as severe as for eBeam inactivated cells, as indicated by the DNA tails protruding from some cells. Nonetheless, the DNA damage in heat-killed cells was more pronounced than in the live cells, since not every cell had distinct DNA tails (**Figure 2**).

### Metabolic Activity in eBeam Inactivated *E. coli* Cells

**FIGURE 3:**
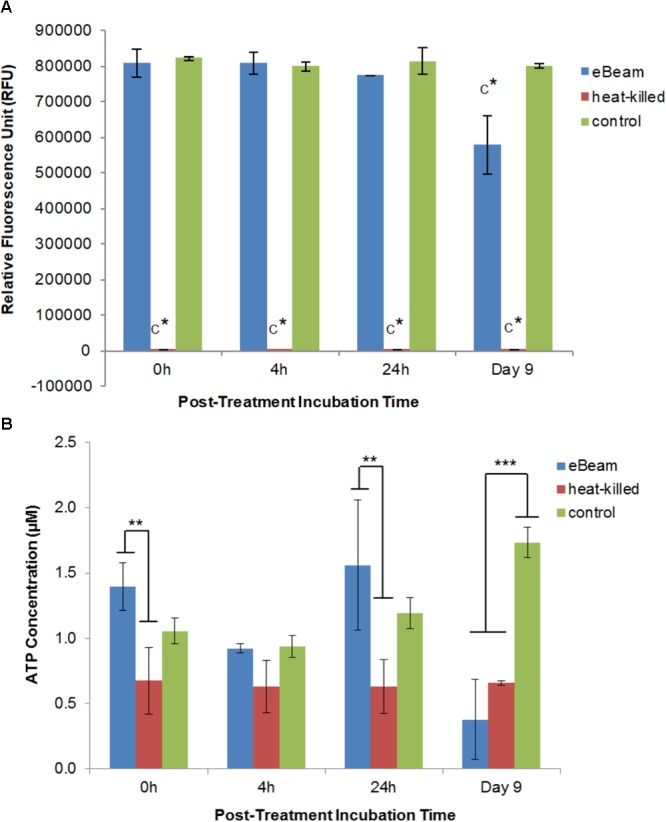
**(A)** Metabolic activity of eBeam inactivated, heat-killed, and live *E. coli* cells. Heat-killed cells did not have measurable levels. Bacterial cultures were incubated at 4°C in LB broth post-treatment and measurements were taken at 0, 4, 24 h, and 9 days. Two independent experiments were performed, with standard deviations shown. C^∗^ denotes statistical significance (*p* < 0.0001). **(B)** ATP levels of eBeam inactivated, heat-killed, and non-treated *E. coli* cells. Samples were incubated at 4°C in LB broth post-treatment and measurements were taken at 0, 4, 24 h, and 9 days. Two independent experiments were performed, with standard deviations shown. ^∗∗^Denotes statistical significance (*p* < 0.01); ^∗∗∗^denotes statistical significance (*p* < 0.001).

Live *E. coli* cells maintained a high level of metabolic activity over the entire 9 day incubation period (LB broth at 4°C), whereas heat-killed cells exhibited no metabolic activity (**Figure 3A**). In fact, the heat-killed cells were significantly different (*p* < 0.0001) from eBeam inactivated and live cells (**Figure 3A**). Metabolic activity in eBeam inactivated *E. coli* cells was maintained at levels comparable to the live cells over a period of 24 h post-irradiation. By day 9, the metabolic activity in the eBeam inactivated cells had significantly (*p* < 0.0001) decreased compared to the live cells (**Figure 3A**).

### ATP Levels in eBeam Inactivated *E. coli* Cells

The ATP levels for the live *E. coli* cells increased over the 9 day incubation period (0 h: 1.06 μM; 4 h: 0.94 μM; 24 h: 1.19 μM; day 9: 1.73 μM) (**Figure 3B**). In contrast, heat-killed cultures maintained constant ATP levels throughout the entire 9 day incubation period (0 h: 0.67 μM; 4 h: 0.63 μM; 24 h: 0.63 μM; day 9: 0.66 μM) (**Figure 3B**). ATP levels for eBeam inactivated *E. coli* cells were much more variable compared to heat-killed and live cells (0 h: 1.4 μM; 4 h: 0.92 μM; 24 h: 1.56 μM; day 9: 0.38 μM) (**Figure 3B**). At 0 h, the eBeam inactivated cells had the highest ATP levels compared to live and heat-killed cells. In addition, the ATP levels were significantly different (*p* < 0.0062) from the heat-killed cells. At 4 h, all three groups had very similar ATP levels. At 24 h, eBeam inactivated cells had the highest ATP levels and heat-killed cells the lowest. The ATP levels in the eBeam inactivated cells were significantly different (*p* < 0.0011) from the heat-killed cells. After 9 days of incubation at 4°C, eBeam inactivated cells had the lowest levels of ATP and the live cells the highest and eBeam inactivated and heat-killed cells had ATP levels that were significantly different (*p* < 0.0001 and *p* < 0.0003, respectively) from the live cells (**Figure 3B**).

### Bacteriophage Multiplication in eBeam Inactivated *E. coli* Cells

**FIGURE 4:**
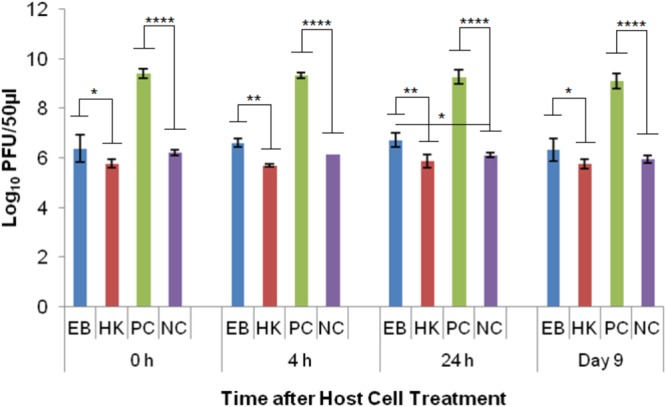
Bacteriophage λ numbers after incubation (at 37°C for 24 h) with eBeam inactivated host cells (EB), heat-killed host cells (HK), non-treated live host cells (PC – positive control), and no host cells (NC – negative control). The 0, 4, 24 h, and day 9 time points represent the time after host cell treatment. Two independent experiments were performed, with standard deviations shown. ^∗^Denotes statistical significance (*p* < 0.05); ^∗∗^denotes statistical significance (*p* < 0.01); ^∗∗∗∗^denotes statistical significance (*p* < 0.0001).

Phage λ was able to reproduce in healthy *E. coli* host cells (PC) as indicated by the significant difference (*p* < 0.0001) to the no host cell negative control (NC) at every time point. The average log PFU increase was 3.18 ± 0.02 across all the time points (**Figure 4**). Phage λ was able to propagate in eBeam inactivated (EB) host cells that were incubated for 24 h post-irradiation (in LB broth at 4°C) (**Figure 4**). At this time point, a statistically significant difference (*p* < 0.05) based on a log PFU increase of 0.61 was observed between EB and NC. At the other 3 time points (0 h, 4 h, and 9 days), there was no statistically significant difference between the PFU counts for phage λ incubated with EB cells and no host cells (NC). However, a slight increase in log PFU numbers (ca. 0.3) was observed at these three time points (**Figure 4**). Phage λ was not able to reproduce in heat-killed (HK) host cells. In fact, a 0.3 log reduction in PFU counts was observed at all four time points (**Figure 4**). A significant difference (*p* < 0.05) was observed between eBeam inactivated and heat-killed host cells at every time point (**Figure 4**).

**FIGURE 5:**
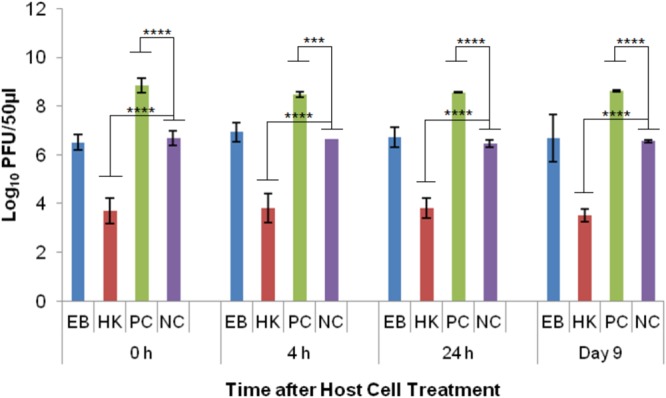
T4 bacteriophage numbers after incubation (at 37°C for 24 h) with eBeam inactivated host cells (EB), heat-killed host cells (HK), non-treated live host cells (PC – positive control), and no host cells (NC – negative control). The 0, 4, 24 h, and day 9 time points represent the time after host cell treatment. Two independent experiments were performed, with standard deviations shown. ^∗∗∗^Denotes statistical significance (*p* < 0.001); ^∗∗∗∗^denotes statistical significance (*p* < 0.0001).

Phage T4D was able to reproduce in healthy *E. coli* host cells (PC) as indicated by the significant difference (*p* < 0.001) to the no host cell control (NC) at every time point. The average log PFU increase was 2.04 ± 0.15 across all the time points (**Figure 5**). Phage T4D numbers in eBeam inactivated host cells (EB) remained at the same levels as the NC for all the time points, indicating that T4D was unable to propagate in EB cells (**Figure 5**). Heat-killed host cells (HK) turned out to be a net sink for T4D phages, reducing its numbers by 2.88 logs on average, as indicated by the significant difference (*p* < 0.0001) to the no host cell (NC) control (**Figure 5**).

**FIGURE 6:**
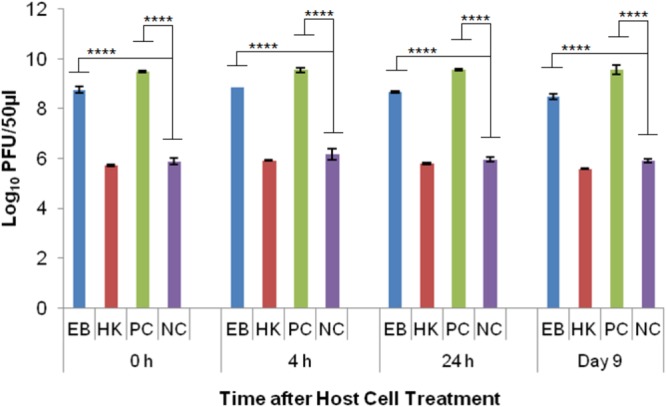
T7 bacteriophage numbers after incubation (at 37°C for 24 h) with eBeam inactivated host cells (EB), heat-killed host cells (HK), non-treated live host cells (PC – positive control), and no host cells (NC – negative control). The 0, 4, 24 h, and day 9 time points represent the time after host cell treatment. Two independent experiments were performed, with standard deviations shown. ^∗∗∗∗^Denotes statistical significance (*p* < 0.0001).

Phage T7 was able to reproduce in healthy *E. coli* host cells (PC) as indicated by the significant difference (*p* < 0.0001) to the no host cell control (NC) at every time point. The average log PFU increase was 3.57 ± 0.15 across all the time points (**Figure 6**). Phage T7 was able to produce progeny particles in eBeam inactivated host cells (EB) at every time point (0, 4, 24 h, and 9 days post-irradiation) (**Figure 6**). Phage T7 numbers in EB cells were significantly different (*p* < 0.0001) from the no host cell control (NC), increasing by at least 2.6 logs at every time point (**Figure 6**). There was no significant difference between the T7 phage numbers in heat-killed host cells (HK) compared to the NC, indicating that T7 phages were unable to propagate in HK host cells (**Figure 6**). However, HK host cells were not a net sink for T7 phages as they were for T4 phages.

## Discussion

### Membrane Integrity of eBeam Inactivated *E. coli* Cells

The vast majority of eBeam inactivated *E. coli* cells maintained their membrane integrity up to 24 h post-irradiation when kept in LB broth at 4°C. These results are congruent with a study by Jesudhasan et al. (2015), which found that a large majority of *Salmonella* Enteritidis cells had intact membranes after exposure to a lethal 2.5 kGy eBeam dose. Only after 9 days of incubation did the membrane damage in the eBeam inactivated *E. coli* cells become more prevalent (**Figure 1**). This is in stark contrast to heat-killed cells, which showed membrane damage immediately following the heat treatment (**Figure 1**). Our results indicate that eBeam inactivated cells resemble live cells more closely with respect to their membrane integrity than heat-killed cells.

### Visualization of DNA Double-Strand Breaks in eBeam Inactivated *E. coli* Cells

Ionizing radiation is known to cause DNA DSBs (Hutchinson, 1985). DSBs are the most lethal form of DNA damage and most organisms can generally tolerate only a few of them (Krasin and Hutchinson, 1977). To confirm that a lethal eBeam dose results in extensive DNA damage, the neutral comet assay was performed on *E. coli* cells irradiated in LB broth. The fluorescent images obtained from the comet assay showed extensive DSBs in the cells after exposure to lethal eBeam irradiation. This is evident by the complete absence of distinct DNA tails/comets (**Figure 2**). The extensive DNA damage in eBeam inactivated cells makes the quantification of DSBs extremely difficult. On the other hand, live healthy *E. coli* cells showed only minor DNA damage as seen by a few long DNA tails, while heat-killed cells exhibited both patterns (**Figure 2**). The lack of distinct DNA tails in the eBeam inactivated cells is a result of the large number of DSBs. It has been estimated that 100 Gy of ionizing radiation cause approximately 1 DSB per one million base pairs (Mbp) (Daly et al., 2010). For the genome of *E. coli* strain K-12 (4.6 Mbp) this translates roughly to 3–5 DSBs per 100 Gy (Blattner et al., 1997; Thomson et al., 2008). Therefore, a dose of 7 kGy would result in 210–350 DSBs per genome. The paper by Singh et al. (1999) is the only other published report that utilized the neutral comet assay to visualize DSBs in irradiated bacteria. They studied x-ray irradiated *E. coli* cells at 0.125–1 Gy and were able to quantify the DNA tails. However, considering the substantial difference in dose, 1 Gy versus 7000 Gy, and the theoretical number of DSBs (0.03 vs. 210 per cell), it is not surprising that distinct or countable DNA tails were not observed in this study. It needs emphasis that DNA comet assays are not particularly useful for prokaryotes because of their low cellular DNA content. Therefore attempting to quantify DNA tails or “spots” would be prone to serious errors. These are key drawbacks of utilizing DNA comet assays in prokaryotes. In preliminary studies using DNA fragmentation analysis, we have observed that DNA fragments in the 10^3^ bp predominate after eBeam irradiation as compared to the 10^4^ bp fragments that are present in un-irradiated cells (*data not included*).

### Metabolic Activity in eBeam Inactivated *E. coli* Cells

eBeam irradiated *E. coli* cells incubated in LB broth at 4°C maintained metabolic activity levels on par with the positive control cells for the first 24 h (**Figure 3A**). This trend was also observed for lethally irradiated *S*. Typhimurium cells incubated in TSB at 4°C. We hypothesize that the lethally irradiated cells are adapting to the cold environment and are adjusting their metabolic needs to focus on DNA repair (Daly and Minton, 1996; Panoff et al., 1998; Liu et al., 2003; Kimura et al., 2006; Dillingham and Kowalczykowski, 2008). By day 9 of incubation the metabolic activity in irradiated cells had decreased significantly compared to the control (**Figure 3A**). This trend could signify the beginning of the cell death phase and is congruent with an observed decrease in membrane integrity (**Figure 1**) (Wanner and Egli, 1990; Finkel, 2006).

### ATP Levels in Lethally Irradiated *E. coli* Cells

ATP levels for eBeam irradiated *E. coli* samples were more variable compared to heat-killed and control samples (**Figure 3B**). In general, irradiated samples resembled control samples more closely than heat-killed ones, except on day 9 of incubation, at which point the ATP levels in lethally irradiated cells had decreased significantly. The observed trend in ATP levels indicates that irradiated cells were metabolically active (to varying degrees) over the 9 day incubation period. These observations together with the results from the redox indicator (alamarBlue^®^) support our hypothesis that lethally irradiated cells remain metabolically active for extended periods of time after irradiation. Similar results were obtained by Magnani et al. (2009) and Secanella-Fandos et al. (2014) with lethally gamma irradiated *Brucella melitensis* and *Mycobacterium bovis* cells, respectively.

### Bacteriophage Multiplication in Lethally Irradiated *E. coli* Cells

All of the three bacteriophages tested, namely λ, T4, and T7, are tailed, double-stranded DNA phages belonging to the order *Caudovirales* (Ackermann, 2006). All three of them require their host cell’s machinery to varying degrees for their propagation. Phage λ relies completely on the host cell to reproduce, T4 requires certain components of the host cell, and T7 only requires the host’s machinery at the very beginning of infection (Hendrix and Casjens, 2006; Molineux, 2006; Mosig and Eiserling, 2006).

Phage λ is most dependent on its host cell and it also has one of the best-understood complex regulatory systems. λ is a temperate phage, with an ability to choose between two alternative life styles: the lytic and the lysogenic growth cycles. The decision between the two cycles is made within the first 10–15 min of infection and depends both on the MOI and the physiological state of the host cell (Little, 2006). λ uses the energy of the host cell’s metabolism and its biosynthetic machinery to produce ca. 50–100 progeny virions (Little, 2006). Cell lysis occurs after ca. 1 h of infection in healthy host cells (Little, 2006). The results of this study showed that at the 24 h time point (post-irradiation), there was a statistically significant difference (*p* < 0.05) between eBeam irradiated host cells and the no host cell control (**Figure 4**), indicating that phage λ was able to propagate successfully in eBeam irradiated cells. A similar trend was observed for the remaining three time points (0 h, 4 h, and day 9), but the increase in virus numbers was not statistically significant (**Figure 4**). Since λ phages are able to propagate inside eBeam irradiated *E. coli* host cells, we hypothesize that all of the necessary cellular resources/machineries are still functioning within the irradiated cells. Phage λ requires the host’s (1) RNA polymerase for all its transcription needs, (2) entire DNA replication apparatus for its phage DNA replication, and (3) translation machinery to make its proteins (Baranska et al., 2001; Hendrix and Casjens, 2006). All of these cellular functions must still be in “good working order” in eBeam irradiated cells; otherwise phage λ would not be able to propagate. It is possible that λ used the host cell’s pre-formed RNA polymerase as well as other macromolecules to carry out the transcription and translation of its DNA. Whether or not pre-formed molecules were used, these results prove that the host cell’s RNA polymerase is able to transcribe DNA and the ribosomes are able to translate and make proteins after a lethal eBeam irradiation dose.

The results for the T4 phage experiments revealed that they were unable to propagate in eBeam irradiated host cells. Interestingly, heat-killed host cells were a net sink for T4 phages, reducing phage numbers by approximately 3 logs (**Figure 5**). T4 phages also depend on many vital host structures and functions, such as membranes, energy metabolism, transcriptional and translational machines, and some chaperones (Adelman et al., 1997; Bhagwat and Nossal, 2001; Mosig and Eiserling, 2006). T4 phages use and modify the core host RNA polymerase, through phage-induced proteins, to selectively transcribe the hydroxymethylcytosine (HMC) residue containing phage DNA rather than the cytosine residue containing host DNA (Drivdahl and Kutter, 1990; Kashlev et al., 1993; Mosig and Eiserling, 2006). In fact, all host DNA and mRNA present at the time of infection, are rapidly degraded and the breakdown products are used to synthesize phage DNA and RNA. Furthermore, after infection, the translation of host messages ceases and ribosomes are re-programmed to translate T4 messages (Duckworth, 1970). Other than phage λ, T4 phage codes for all the components of its own DNA replication and recombination complexes (Mosig, 1998; Mosig et al., 1998; Kolesky et al., 1999; Mosig and Eiserling, 2006). It is unclear which structural or functional component(s) of the eBeam irradiated host cells were not functioning properly to prevent T4 propagation. It is possible that all of the host cell modifications (i.e., RNA polymerase) initiated by T4 phages increased the overall oxidative stress within the host cells, rendering them ineffective for phage propagation. Krisko and Radman (2010) found that the decline of what they called biosynthetic efficacy (measured by λ propagation) was correlated to radiation-induced oxidative damage. Targeted studies are needed to address this issue as well as the sink phenomenon observed in heat-killed host cells (**Figure 5**).

The results for the T7 phage experiments showed that they were the most successful in utilizing eBeam irradiated host cells for their propagation out of all the phages tested (**Figure 6**). T7 growth is remarkably independent of host enzymes; it only requires the host’s translational apparatus and biosynthetic machinery for precursor synthesis (Molineux, 2006). The host cell’s RNA polymerase is used to make early RNAs, but most of the transcription is catalyzed by the T7 RNA polymerase (once it has been synthesized by the host cell). T7 DNA replication and recombination are also independent of host proteins, except for thioredoxin (Martin et al., 1988; Imburgio et al., 2000; Molineux, 2006). Just like T4 phages, T7 phages attach to the lipopolysaccharides of the outer membrane and translocate their DNA via a self-made channel into the host cell’s cytoplasm. DNA translocation is highly temperature dependent and requires membrane potential (Molineux, 2001, 2006). Since T7 phages require the least amount of host cell resources and functionalities, this may be the reason why they were able to propagate so efficiently in eBeam irradiated host cells (**Figure 6**). Furthermore, the results indicate that all of the cellular components (i.e., RNA polymerase) needed by the phage to replicate are functioning properly in eBeam irradiated host cells. It would further appear that irradiated cells kept at 4°C post-irradiation are “frozen in time” (in terms of their cellular activities), since T7 phages were able to propagate in cells that had been stored for 9 days just as well as in freshly irradiated cells (**Figure 6**). This is in contrast to post-irradiation incubation at 37°C. Marsden et al. (1972) found that sub-lethally irradiated *E. coli* cells rapidly lost their ability to support T4 phage growth after 2 h of post-irradiation incubation at 37°C. Even though, T7 phages were able to propagate well in irradiated cells, the increase in numbers was still significantly different (*p* < 0.0001) from the non-irradiated (control) cells (**Figure 6**). This leads to the conclusion that some cellular components, apart from the DNA, were rendered less functional due to the irradiation with a lethal eBeam dose. These results are in line with earlier studies that examined phage growth in x-ray irradiated *E. coli* host cells (Labaw et al., 1953; Pollard et al., 1958). The results presented here with eBeam irradiated *E. coli* host cells have raised many more interesting questions (i.e., do phages use pre-formed or newly synthesized RNA polymerase) and warrant further investigation. Using bacteriophages to investigate the functionality of lethally irradiated bacterial cells may prove to be a very elegant model system.

## Conclusion

The results presented indicate that lethally irradiated *E. coli* cells resemble live (non-irradiated) cells more closely than heat-killed cells. Despite their extensive DNA damage, lethally irradiated cells have intact membranes, are metabolically active, and are able to support the propagation of bacteriophages.

## Author Contributions

A-SH and SP conceived the experiments and wrote the manuscript. A-SH performed the experiments and data analysis.

## Conflict of Interest Statement

The authors declare that the research was conducted in the absence of any commercial or financial relationships that could be construed as a potential conflict of interest.

## References

[B1] Ackermann H.-W. (2006). “Classification of bacteriophages,” in *The Bacteriophages* ed. Calendar R. (New York, NY: Oxford University Press) 8–16.

[B2] Adams M. H. (1959). *Bacteriophages.* New York, NY: Interscience Publishers, Inc.

[B3] Adelman K. Orsini G. Kolb A. Graziani L. Brody E. N. (1997). The interaction between the AsiA protein of bacteriophage T4 and the sigma(70) subunit of *Escherichia coli* RNA polymerase. *J. Biol. Chem.* 272 27435–27443. 10.1074/jbc.272.43.27435 9341196

[B4] Baranska S. Gabig M. Wegrzyn A. Konopa G. Herman-Antosiewicz A. Hernandez P. (2001). Regulation of the switch from early to late bacteriophage lambda DNA replication. *Microbiology* 147 535–547. 10.1099/00221287-147-3-535 11238961

[B5] Bhagwat M. Nossal N. G. (2001). Bacteriophage T4 RNase H removes both RNA primers and adjacent DNA from the 5′ end of lagging strand fragments. *J. Biol. Chem.* 276 28516–28524. 10.1074/jbc.M103914200 11376000

[B6] Blattner F. R. Plunkett G. III Bloch C. A. Perna N. T. Burland V. Riley M. (1997). The complete genome sequence of *Escherichia coli* K-12. *Science* 277 1453–1462. 10.1126/science.277.5331.1453 9278503

[B7] Daly M. J. Gaidamakova E. K. Matrosova V. Y. Kiang J. G. Fukumoto R. Lee D. Y. (2010). Small-molecule antioxidant proteome shields in *Deinococcus radiodurans*. *PLoS One* 5:e12570. 10.1371/journal.pone.0012570 20838443 PMC2933237

[B8] Daly M. J. Gaidamakova E. K. Matrosova V. Y. Vasilenko A. Zhai M. Leapman R. D. (2007). Protein oxidation implicated as the primary determinant of bacterial radioresistance. *PLoS Biol.* 5:e92. 10.1371/journal.pbio.0050092 17373858 PMC1828145

[B9] Daly M. J. Minton W. K. (1995a). Interchromosomal recombination in the extremely radioresistant bacterium *Deinococcus radiodurans*. *J. Bacteriol.* 177 5495–5505. 10.1128/jb.177.19.5495-5505.1995 7559335 PMC177357

[B10] Daly M. J. Minton K. W. (1995b). Resistance to radiation. *Science* 270 1318–1318. 10.1126/science.270.5240.1318 7481818

[B11] Daly M. J. Minton K. W. (1996). An alternative pathway of recombination of chromosomal fragments precedes recA-dependent recombination in the radioresistant bacterium *Deinococcus radiodurans*. *J. Bacteriol.* 178 4461–4471. 10.1128/jb.178.15.4461-4471.1996 8755873 PMC178212

[B12] Daly M. J. Minton K. W. (1997). Recombination between a resident plasmid and the chromosome following irradiation of the radioresistant bacterium *Deinococcus radiodurans*. *Gene* 187 225–229. 10.1016/S0378-1119(96)00755-X 9099885

[B13] Dillingham M. S. Kowalczykowski S. C. (2008). RecBCD enzyme and the repair of double-stranded DNA breaks. *Microbiol. Mol. Biol. Rev.* 72 642–671. 10.1128/MMBR.00020-08 19052323 PMC2593567

[B14] Dorpema J. W. (1990). Review and state-of-the-art on radiation sterilization of medical devices. *Radiat. Phys. Chem.* 35 357–360. 10.1016/1359-0197(90)90118-2 6761276

[B15] Drivdahl R. H. Kutter E. M. (1990). Inhibition of transcription of cytosine-containing DNA in vitro by the alc gene product of bacteriophage T4. *J. Bacteriol.* 172 2716–2727. 10.1128/jb.172.5.2716-2727.1990 2185231 PMC208917

[B16] Duckworth D. H. (1970). Biological activity of bacteriophage ghosts and take-over of host functions by bacteriophage. *Bacteriol. Rev.* 34 344–363. 4918524 10.1128/br.34.3.344-363.1970 PMC378358

[B17] Farkas J. Mohacsi-Farkas C. (2011). History and future of food irradiation. *Trends Food Sci. Tech.* 22 121–126. 10.1016/j.tifs.2010.04.002

[B18] Finkel S. E. (2006). Long-term survival during stationary phase: evolution and the GASP phenotype. *Nat. Rev. Microbiol.* 4 113–120. 10.1038/nrmicro1340 16415927

[B19] Follett P. A. (2002). Mango seed weevil (Coleoptera: Curculionidae) and premature fruit drop in mangoes. *J. Econ. Entomol.* 95 336–339. 10.1603/0022-0493-95.2.336 12020010

[B20] Hendrix R. W. Casjens S. (2006). “Bacteriophage λ and its genetic neighborhood,” in *The Bacteriophages* ed. Calendar R. (New York, NY: Oxford University Press) 409–447.

[B21] Holloman W. K. Schirawski J. Holliday R. (2007). Towards understanding the extreme radiation resistance of *Ustilago maydis*. *Trends Microbiol.* 15 525–529. 10.1016/j.tim.2007.10.007 17997098

[B22] Hutchinson F. (1985). Chemical changes induced in DNA by ionizing radiation. *Prog. Nucleic Acid Res. Mol. Biol.* 32 115–154. 10.1016/S0079-6603(08)60347-5 3003798

[B23] Imburgio D. Rong M. Ma K. McAllister W. T. (2000). Studies of promoter recognition and start site selection by T7 RNA polymerase using a comprehensive collection of promoter variants. *Biochemistry* 39 10419–10430. 10.1021/bi000365w 10956032

[B24] Jesudhasan P. R. McReynolds J. L. Byrd A. J. He H. Genovese K. J. Droleskey R. (2015). Electron-beam-inactivated vaccine against *Salmonella* Enteritidis colonization in molting hens. *Avian Dis.* 59 165–170. 10.1637/10917-081014-ResNoteR 26292553

[B25] Kashlev M. Nudler E. Goldfarb A. White T. Kutter E. (1993). Bacteriophage T4 Alc protein - a transcription termination factor sensing local modification of DNA. *Cell* 75 147–154. 10.1016/S0092-8674(05)80091-1 8402894

[B26] Kimura S. Ishidou E. Kurita S. Suzuki Y. Shibato J. Rakwal R. (2006). DNA microarray analyses reveal a post-irradiation differential time-dependent gene expression profile in yeast cells exposed to x-rays and gamma-rays. *Biochem. Biophys. Res. Commun.* 346 51–60. 10.1016/j.bbrc.2006.05.126 16759639

[B27] Kolesky S. Ouhammouch M. Brody E. N. Geiduschek E. P. (1999). Sigma competition: the contest between bacteriophage T4 middle and late transcription. *J. Mol. Biol.* 291 267–281. 10.1006/jmbi.1999.2953 10438620

[B28] Krasin F. Hutchinson F. (1977). Repair of DNA double-strand breaks in *Escherichia coli*, which requires RecA function and presence of a duplicate genome. *J. Mol. Biol.* 116 81–98. 10.1016/0022-2836(77)90120-6 338918

[B29] Krisko A. Radman M. (2010). Protein damage and death by radiation in *Escherichia coli* and *Deinococcus radiodurans*. *Proc. Natl. Acad. Sci. U.S.A.* 107 14373–14377. 10.1073/pnas.1009312107 20660760 PMC2922536

[B30] Labaw L. W. Mosley V. M. Wyckoff R. W. G. (1953). Development of bacteriophage in x-ray inactivated bacteria. *J. Bacteriol.* 65 330–336. 13034747 10.1128/jb.65.3.330-336.1953 PMC169527

[B31] Lemay M. Wood K. A. (1999). Detection of DNA damage and identification of UV-induced photoproducts using the CometAssay kit. *Biotechniques* 27 846–851. 10.2144/99274pf01 10524327

[B32] Little J. W. (2006). “Gene regulatory circuitry of phage λ,” in *The Bacteriophages* ed. Calendar R. (New York, NY: Oxford University Press) 74–82.

[B33] Liu Y. Q. Zhou J. Omelchenko M. V. Beliaev A. S. Venkateswaran A. Stair J. (2003). Transcriptome dynamics of *Deinococcus radiodurans* recovering from ionizing radiation. *Proc. Natl. Acad. Sci. U.S.A.* 100 4191–4196. 10.1073/pnas.0630387100 12651953 PMC153069

[B34] Magnani D. M. Harms J. S. Durward M. A. Splitter G. A. (2009). Nondividing but metabolically active gamma-irradiated Brucella melitensis is protective against virulent B. melitensis challenge in mice. *Infect. Immun.* 77 5181–5189. 10.1128/IAI.00231-09 19703982 PMC2772552

[B35] Makarova K. S. Omelchenko M. V. Gaidamakova E. K. Matrosova V. Y. Vasilenko A. Zhai M. (2007). *Deinococcus geothermalis*: the pool of extreme radiation resistance genes shrinks. *PLoS One* 2:e955. 10.1371/journal.pone.0000955 17895995 PMC1978522

[B36] Marsden H. Ginoza W. Pollard E. C. (1972). Effects of ionizing radiation on capacity of *Escherichia coli* to support bacteriophage T4 growth. *J. Virol.* 9 1004–1016. 4556509 10.1128/jvi.9.6.1004-1016.1972 PMC356407

[B37] Martin C. T. Muller D. K. Coleman J. E. (1988). Processivity in early stages of transcription by T7 RNA polymerase. *Biochemistry* 27 3966–3974. 10.1021/bi00411a012 3415967

[B38] Miyahara M. Miyahara M. (2002). Effects of gamma ray and electron beam irradiations on survival of anaerobic and facultatively anaerobic bacteria. *Kokuritsu Iyakuhin Shokuhin Eisei Kenkyusho Hokoku* 120 75–80. 12638185

[B39] Molineux I. J. (2006). “The T7 group,” in *The Bacteriophages* ed. Calendar R. (New York, NY: Oxford University Press) 277–301.

[B40] Molineux L. J. (2001). No syringes please, ejection of phage T7 DNA from the virion is enzyme driven. *Mol. Microbiol.* 40 1–8. 10.1046/j.1365-2958.2001.02357.x 11298271

[B41] Mosig G. (1998). Recombination and recombination-dependent DNA replication in bacteriophage T4. *Annu. Rev. Genet.* 32 379–413. 10.1146/annurev.genet.32.1.379 9928485

[B42] Mosig G. Colowick N. E. Pietz B. C. (1998). Several new bacteriophage T4 genes, mapped by sequencing deletion endpoints between genes 56 (dCTPase) and dda (a DNA-dependent ATPase-helicase) modulate transcription. *Gene* 223 143–155. 10.1016/S0378-1119(98)00238-8 9858714

[B43] Mosig G. Eiserling F. (2006). “T4 and related phages: structure and development,” in *The Bacteriophages* ed. Calendar R. (New York, NY: Oxford University Press) 225–267.

[B44] Nakayama G. R. Caton M. C. Nova M. P. Parandoosh Z. (1997). Assessment of the alamar blue assay for cellular growth and viability in vitro. *J. Immunol. Methods* 204 205–208. 10.1016/S0022-1759(97)00043-4 9212838

[B45] Ostling O. Johanson K. J. (1984). Microelectrophoretic study of radiation-induced DNA damages in individual mammalian cells. *Biochem. Biophys. Res. Commun.* 123 291–298. 10.1016/0006-291X(84)90411-X 6477583

[B46] Panoff J. M. Thammavongs B. Guéguen M. Boutibonnes P. (1998). Cold stress responses in mesophilic bacteria. *Cryobiology* 36 75–83. 10.1006/cryo.1997.2069 9527869

[B47] Pillai S. D. McElhany K. (2011). Status of food irradiation in the USA. *Safe Food* 6 1–10.

[B48] Pillai S. D. Shayanfar S. (2015). “Introduction to electron beam pasteurization in food processing,” in *Electron Beam Pasteurization and Complementary Food Processing Technologies* eds Pillai S. D. Shayanfar S. (Cambridge: Woodhead Publishing) 3–10. 10.1533/9781782421085.1.3

[B49] Pollard E. Setlow J. Watts E. (1958). The effect of ionizing radiation on the capacity of bacteria to sustain phage growth. *Radiat. Res.* 8 77–91. 10.2307/3570538 13506037

[B50] Praveen C. Jesudhasan P. R. Reimers R. S. Pillai S. D. (2013). Electron beam inactivation of selected microbial pathogens and indicator organisms in aerobically and anaerobically digested sewage sludge. *Bioresour. Technol.* 144 652–657. 10.1016/j.biortech.2013.07.034 23907065

[B51] Purdie J. W. Ebert M. Tallentire A. (1974). Increased response of anoxic *Bacillus megaterium* spores to radiation at high dose rates. *Int. J. Radiat. Biol.* 26 435–443. 4216588 10.1080/09553007414551461

[B52] Rampersad S. N. (2012). Multiple applications of alamar blue as an indicator of metabolic function and cellular health in cell viability bioassays. *Sensors* 12 12347–12360. 10.3390/s120912347 23112716 PMC3478843

[B53] Secanella-Fandos S. Noguera-Ortega E. Olivares F. Luquin M. Julián E. (2014). Killed but metabolically active *Mycobacterium bovis* bacillus Calmette-Guerin retains the antitumor ability of live bacillus Calmette-Guerin. *J. Urol.* 191 1422–1428. 10.1016/j.juro.2013.12.002 24333111

[B54] Singh N. P. Stephens R. E. Singh H. Lai H. (1999). Visual quantification of DNA double-strand breaks in bacteria. *Mutat Res* 429 159–168. 10.1016/S0027-5107(99)00124-4 10526201

[B55] Squatrito R. C. Connor J. P. Buller R. E. (1995). Comparison of a novel redox dye cell growth assay to the ATP bioluminescence assay. *Gynecol. Oncol.* 58 101–105. 10.1006/gyno.1995.1190 7789873

[B56] Thomson N. R. Clayton D. J. Windhorst D. Vernikos G. Davidson S. Churcher C. (2008). Comparative genome analysis of *Salmonella* Enteritidis PT4 and *Salmonella* Gallinarum 287/91 provides insights into evolutionary and host adaptation pathways. *Genome Res.* 18 1624–1637. 10.1101/gr.077404.108 18583645 PMC2556274

[B57] Wanner U. Egli T. (1990). Dynamics of microbial growth and cell composition in batch culture. *FEMS Microbiol. Lett.* 75 19–44. 10.1111/j.1574-6968.1990.tb04084.x 2186759

[B58] Weiss H. Epp E. R. Heslin J. M. Ling C. C. Santomasso A. (1974). Oxygen depletion in cells irradiated at ultra high dose rates and at conventional dose rates. *Radiat. Res.* 59 247–248. 10.1080/09553007414550901 4607987

